# Thoughts on the Difficulty of Acquiring Accurate Knowledge in Practical Medicine: With Special Reference to the French Schools; Being an Extract from the Inaugural Address before the Medical Society of Tennessee

**Published:** 1845-11

**Authors:** A. H. Buchanan

**Affiliations:** President of the Society


### WESTERN JOURNAL

OF

### MEDICINE AND SURGERY.

---

NOVEMBER, 1845.

---

ART. I.—*Thoughts on the Difficulty of acquiring accurate Knowledge in Practical Medicine, with special reference to the French Schools;* being an extract from the Inaugural Address before the Medical Society of Tennessee. By A. H. BUCHANAN, M.D., President of the Society.

The science of medicine as taught in the modern French Schools, I am persuaded, although well calculated to elicit truth and establish facts in morbid anatomy and pathology, is nevertheless too exclusively devoted to the study of these branches of the science, to the neglect, in a great degree, of practical medicine, the advancement of which should be the principal, as it is the most important object of medical research; and that although the minute and laborious investigations of the morbid anatomy of the body, pursued with so much energy by the French medical philosophers, have established a more exact and accurate knowledge of the true pathology of disease, that it is yet exceedingly questionable whether we can derive the true pathology of disease from post-mortem examinations alone. We may establish the amount and character of disorganization, which occurs in each and every tissue of the body, when any does occur, but a knowledge of such organic lesions as may be noticed does not instruct us in the change which occurs in the vital phenomena as consequences upon such lesions, for we sometimes observe these changes in the vital phenomena, without correspondent lesions to account for them. And these changes in the vital phenomena are said by the philosophers of the old creed to occur anterior to any change in the organization, and consequently, the changes in the organization are said to be the mere effects of diseased action in the vital properties of the part, and therefore, that we cannot expect to find any thing more from the examination of the dead body than a knowledge of the mere effects of the diseased action upon the organization, that, in no case can we by an examination of the lesions of structure, say what were the symptoms or changes in the vital phenomena that accompanied them. But on the other hand it is answered, can we by an examination of the changes in the vital phenomena alone, say what has been the consequences of such change upon the organization? Can we, by any observation of the symptoms of disease or change in the vital phenomena, or by any *a priori* reasoning anticipate the number, extent, and character of the lesions that have occurred in the various vital organs? We presume no one will say we can. The question then presents itself; is it necessary in order to obtain a true knowledge of pathology, that we shall study both the changes in the vital phenomena, and also the changes in the organic structure, which are found in connection with the vital changes; or in other words, both anatomical and physiological pathology? We are really of opinion that it is only by this mode of investigation that we can arrive at truth in pathology. Now then, what is the philosophy of the two great medical schools, to say nothing of the thousand minor sects, who have no settled opinions?

The school of Louis, or "the numerical system," as it has been called, being that which has given rise to the harshest and most extensive criticisms, and which has exercised the greatest influence on the medical world, presents a fair specimen of the modern or French mode of investigating disease. In this system, and in all his pathological researches, although Louis connects the symptoms with the changes which occur in the organization, and leaves his readers at liberty to draw their own conclusions, yet it is very evident that the tenor of his philosophy and investigations, is to establish the nature of disease or pathology, from an examination of organic lesions, independent of the vital changes or symptoms that accompanied them, and that he attaches more importance to the organic lesions which are noticed, than he does to the vital changes; he makes the vital changes consequences or symptoms of organic changes, and makes pathology consist almost exclusively in changes of structure; so that he would rely more upon the similarity of the organic lesions that might occur in a given number of cases to establish their identity, than he would upon any similarity that might exist in the symptoms or vital changes. In other words, he would look more to morbid anatomy than he would to morbid physiology to establish the identity of disease.

Now, the other class believing that disease consists in a change of the vital properties of the organs, independent of any change in the organization, look only to the changes which occur in the vital phenomena as evidence of its existence and character. In other words, the changes in the vital phenomena (or the symptoms) constitute the disease, and the changes in the organization are mere consequences of disease, and therefore, an investigation of these changes or the study of morbid anatomy (say they,) cannot enlighten us as to the true pathology of disease. They argue, that disease must be studied in the living body and not in the dead, and that disease consists in an alteration of vital actions, and not in a change of structure, and that no knowledge of this alteration of structure obtained by morbid anatomy will teach us the nature of the change in the vital actions.

This last philosophy, as well as the former, is to my mind too exclusive, for we never see life except in connection with organization, nor organization unconnected with life; a change then in the vitality of a part cannot occur without producing a change in the organization, they are inseparably connected and when the one is affected the other must be also. We cannot have disease then unconnected with organization, and in the study of pathology we must examine, not only the changes in the vital phenomena, but also those in the organic structure; we cannot arrive at truth otherwise than by this mode of investigation. To say that no light is thrown upon the pathology of disease by the study of morbid anatomy is not true. We do learn by the study of morbid anatomy in what tissue or tissues the disease existed, and what changes have occurred in those tissues. And these changes in the organic structure which we observe are as positive evidence of disease consisting in organic lesions, as any changes which occur in the vital phenomena are evidence of disease consisting in a change of the vital properties. We always see these changes simultaneously, they co-exist and there is about the same propriety in saying that the alterations which we notice in the organic structure are the effects or consequences of disease as there is in saying, that the changes in the vital phenomena are effects (or symptoms) of disease. The truth is, that disease consists in a simultaneous change of the organic structure and of the vital properties of the structure, and there can be no disease without organic lesion and also a change in the vital properties.

Now, during life the changes which occur in the vital phenomena are evident to our observation, and are what are commonly called symptoms of disease, but the organic lesions in the vital organs are hidden from our view and we cannot observe the changes which are taking place. The object therefore of pathological investigation is to correct the changes which we notice in the vital phenomena with those which we know must exist somewhere in the organism, and true pathology consists in a knowledge of the changes which we see in the vital phenomena in connection with those that exist in the organic structure during life. It does not consist as the vitalists contend in a change of the vital properties alone, which we observe during life; nor as the morbid anatomists contend in the organic lesions which we observe after death. But to connect these changes which we observe in the vital phenomena with those which occur in the organic structure, so as to diagnosticate with certainty the nature and seat of the organic lesion, is one of the most difficult propositions in medicine.

The mode of investigation adopted and pursued with so much zeal by the French pathologists, and apparently with as much caution and strict regard to truth as the nature of the subject would admit of, has done much to advance our knowledge of the pathology of many diseases; yet there is much more to be done even in regard to those which have been most strictly investigated. The intrinsic difficulty of the subject growing out of the thousand modifying circumstances which belong to each case, as the difference in age, sex, original constitution, mode of life, influence of climate and occupation, the time the patient is first examined after the attack; the effects of remedies, the almost constant variation of the symptoms, growing out of the continuance of the disease and its extension to different vital organs, and various other influences are all so many difficulties to be encountered and considered in the investigation of each case; and the consideration of the fact, that the same symptoms occur in connection with different organic lesions, and different symptoms with the same or apparently the same lesions, renders it almost impossible to arrive at rigorous facts or conclusions respecting any disease.

It is the consideration of the fact, that the same lesions do not always give rise to the same symptoms, that has induced the vitalists to contend that disease does not consist in organic lesions; but in a change of the vital phenomena or symptoms alone; but it is a bad rule that will not work both ways, for it may be well asked, why do not the same symptoms always indicate the same disease, and produce the same organic lesions? Why do we, in some cases with a certain group of symptoms observe, upon post-mortem examination, organic lesions, and in other cases with the same symptoms no organic lesions, or different organic lesions? This is a question which it is as incumbent upon the vitalists to answer as it is upon the morbid anatomists. Why did not Louis observe the same organic lesions in all the vital organs of all of those who died of the typhoid affection? It is well known that he observed but one lesion which was *constant*, while he observed lesions of various characters in almost all the vital organs, which he said were common to other acute diseases and were not, therefore, characteristic of the typhoid affection, nor by any means constant in the same organs in different cases. Why, we ask, was this the fact, when the changes in the vital phenomena in each case were so identical as to characterise with certainty the typhoid disease? If symptoms depend upon lesions, ought there not to have been as great a diversity of them in each case as there was in the lesions? And if on the other hand, the symptoms were identical, and they alone constituted the disease, ought not the effects, consequences, or lesions of the symptoms to have been the same? Or, if the disease consisted alone in the changes observed in the vital phenomena, why should there be any organic lesions? The organization, they say, is not the seat of disease, but the *vital properties;* why then is the organization affected in any disease? Or why is the *absence* of organic lesions regarded as proof that disease does consist alone in a change of the vital properties? If, upon post-mortem examination of those who died of the typhoid disease (or any other disease) no organic lesions had been found it would have been seized on as positive proof by the vitalists, that disease consisted alone in a change of the vital properties; but since in every case lesions have been found, it is as much incumbent upon them to account for them, and explain their diversity of seat and character, as it is upon the morbid anatomists to account for the same symptoms occurring in connection with different lesions.

Again, when we recognize a disease to be the same in two or more individuals, as for example, the typhoid affection, from the same or similar changes which we observe to exist in the vital phenomena of the different cases; but upon a post-mortem examination we find various organic lesions in one case, which we do not in another case, how can we with any propriety refer these changes in the vital phenomena to the lesions in the organs which we observe after death? This is a question propounded to the morbid anatomists, and one which remained unanswered, until the pathological researches of the French schools, and particularly of Louis, satisfied many minds in regard to it. It was a question that had to be answered. For if the changes in the vital phenomena do not depend upon organic lesions, and cannot be traced to those lesions as causes, so that they may be established as signs of such lesions, then the study of morbid anatomy would be useless. Accordingly we find that Louis, by his extensive pathological researches, has attempted to prove that notwithstanding we find many lesions in the different vital organs after death from the typhoid disease, that yet there is invariably found one lesion located in the glands of Peyer in the small intestines to which we should refer the changes which occur in the vital phenomena in the typhoid disease, and that no other lesion however extensive it may be, or in what organ it may be found, or however numerous may be the organs in which lesions are found, can yet be considered as evidence of the existence of the typhoid affection, because, they have all been noticed in other acute diseases, and consequently they must give rise to the symptoms of other acute diseases, and not to the symptoms of the typhoid disease, the symptoms of which must all be referred to the lesions in the glands of the small intestines, because these are the only lesions which are constantly found. The chills, stupor, delirium, prostration, anorexia, diarrhœa, &c., are all referred to the changes which occur in the glands of Peyer, and according to the law established by the numerical system, as it has been called, if the glands of Peyer are not affected there is no typhoid affection. Now, I must confess, all this is very hard to believe, but how are we to get over it? If this lesion is the only one which is invariably found in the typhoid disease, and if subsequent researches should confirm this fact; and also, if it is the only one which is not common in other diseases, while all the other lesions are of common occurrence in other diseases, it would seem that to this lesion should be referred at least those symptoms which are characteristic of the affection. But it is extremely difficult to say what these symptoms are, although for practical purposes it is absolutely essential that they should be early recognized, and before any secondary lesions arise in the course of the disease, for if we have to wait several days, and until the primary symptoms are modified by the extension of organic lesions among the different vital organs, in order to make out a clear diagnosis, it deprives us of the most favorable time to arrest its progress. We should recognize this disease then and all others, if possible, at an early period by an examination of the vital phenomena in connection with the organic lesion or lesions that produce them; this is the great object to be attained by pathological research.

But the above philosophy of Louis, which refers all the characteristic symptoms of the typhoid affection to the lesions of the glands of Peyer which he considers the characteristic lesion, is not free from objections; for it cannot be denied that those secondary lesions as they are called, however variable they may be in their appearance and locality, are in truth as much the lesions of the typhoid disease as those which appear in the glands of Peyer, and if they have nothing in them to distinguish them from similar lesions which have been noticed in other diseases, it must create a suspicion in any mind that there can be nothing specific in the lesions of the glands of Peyer which characterizes this affection, for the lesions of the liver, the spleen, œsophagus, and stomach when they do occur in the typhoid disease, are certainly as much lesions of the typhoid disease as those which occur in the glands of Peyer; and our inability to distinguish them from similar lesions in other acute diseases only proves that our pathology of diseased structure is defective, and not that these are not as much characteristic of the typhoid disease as those noticed in the glands of Peyer. And further, that Louis must have referred to the glands of Peyer as presenting the specific lesion of the typhoid disease, not because he really discovered any specific modification of diseased action in the altered structure, but simply because this lesion was *invariably* found in this disease, for if he could discover a peculiarity in this lesion, why could he not discover a peculiarity in the the other lesions which must have been equally lesions of the typhoid disease? And again, if symptoms depend upon organic lesions, then the symptoms of this disease must be constantly changing as the organic lesions extend from one organ to another, and consequently it would seem there can be no fixed or constant symptoms that can be relied on as certainly characteristic of the affection. Now this is pretty much the fact, and it is certainly true, that we judge of the existence of the disease more from a general view of the change which we observe in the whole expression and modification of the vital phenomena, than from any specific symptom depending upon any specific lesion. Nor can we conceive how an organic lesion in the glands of Peyer can justly be regarded as the seat of the delirium and stupor, two cerebral symptoms which almost invariably attend the typhoid disease. These are symptoms which depend upon the condition of the brain and must be referred to it as their cause, and if there can be found no lesions in the brain to account for them, it seems to me to be more in accordance with sound philosophy and physiology to refer them to some inappreciable lesions of its structure, than to say they depend upon the lesions found in the glands of Peyer. But the question again recurs: How can we refer the symptoms which characterize the typhoid affection to lesions inappreciable to our senses; or to lesions common to other diseases; without destroying the force of the pathological law, that lesions of organs give rise to symptoms characteristic of their condition, and by which their condition can be ascertained. Surely those lesions common to other diseases cannot produce symptoms characteristic of the typhoid affection, and it will not do to refer them to inappreciable lesions in the brain, when positive lesions are found elsewhere, for this would be denying the importance of pathological anatomy? If then there are found any lesions not common to other diseases, we are forced to refer the symptoms to those lesions, and hence we see Louis refers the specific symptoms to the lesions of the glands of Peyer, and in truth there was no other alternative left him.

Now, I cannot fully concur in the truth of this philosophy any more than I can in that of the vitalists, who, as before remarked, make disease consist exclusively in a change of the vital properties, and in the language of Louis himself it forces upon us the conviction that there is something more in disease than we can see. You see then how extremely difficult it is to arrive at truth in pathology.

It may be, and the facts established by M. Louis seem to justify the conclusion, that the typhoid affection originally commences in the glands of Peyer, but when by sympathy or any other unknown power, the disease is propagated to distant organs, it does not appear philosophical to refer the symptoms which arise in consequence of diseased actions, in these organs, to the original seat of the malady, nor to regard the organic lesions which occur in them as less specific or less characteristic of the peculiar affection, than the original one.

Surely when a child is inocculated with small pox virus any subsequent pustules or lesions which occur on its body are as truly of the same nature and as characteristic of the affection as the one at the point of inoculation, and it cannot be proper to refer the symptoms characteristic of the disease exclusively to the original point of inception, for if this point had been in any other part, the consequences would have been the same, and the virus from any subsequent pustules in the course of the disease would produce upon a third person the same effects as that from the original one. Now, the same must be true as regards the typhoid disease, and any lesions which occur in its progress must be of the same nature as that at the point of inception, and hence we conclude that all lesions which occur in its progress are of the same nature as the original one.

But these are difficulties in the doctrines of the morbid anatomists, which if it is incumbent upon them to explain away, it is not less so for the vitalists to prove that their positions are correct, for how can they say that symptoms depend upon modifications of the vital properties alone when lesions of structure, whether produced mechanically or by the action of morbific agents, are every where known and recognized as the certain causes of the changes which we witness in the vital phenomena. Hear, for example, what one of the most ardent and learned supporters of the vital doctrine says even while criticising with the greatest severity the philosophy of Louis. I allude to Professor Payne in his Physiological Commentaries, vol. 2, p. 432. In speaking of the cerebral symptoms of the typhoid disease, he says—

"And since no such phenomena not even constitutional ones arise when the jugular veins are tied, and *never* unless there be *venous congestion*, or common *inflammation*, or some *organic* affection of the brain, the conclusion appears unavoidable that the cerebral results depended upon a highly morbid condition of the venous tissue of that part in all the cases where venous injection was the only appreciable lesion."

But now let us enquire of our learned author upon what these same cerebral symptoms depended when there was *no* appreciable lesion. He says "never unless there be venous congestion, or common inflammation, or some organic affection of the brain do such symptoms arise." But when Louis tells him he discovered more of these lesions and yet the symptoms existed, and therefore he felt justified in referring them to the lesions of the glands of Peyer, how does he account for their existence? We presume from his doctrine which shews itself upon every page of his work, that he would say in all such cases the symptoms must be referred to the changes in the vital properties of the part. It would seem however, that in his criticisms he came to the "unavoidable conclusion that the cerebral results (or symptoms) depended upon a highly morbid condition of the venous tissue of the part in all the cases where venous congestion was the only appreciable lesion." Here then is a clear admission on the part of one of the ablest and most strenuous advocates of the vital doctrine, that symptoms do depend upon organic lesions, and if they are mere types or signs of disease in one case they may be so in all cases, and hence the doctrine of the morbid anatomist that symptoms are consequences of organic lesions, is not without foundation.

It would be easy to extend the consideration of this difficult and complex study, by noticing the numerous organs of the body, their various relations to each other, and their vital susceptibilities to the thousand causes capable of producing diseased action in them, as well as their special relations or susceptibilities to the action of particular causes, and shew that it should not surprize us that there should exist a diversity of opinion respecting the best mode of cultivating it; or wonder that it is still involved in so much obscurity.

I am well aware that the mode of investigation adopted by the French schools, consisting in a rigid examination, not only of the changes in the vital phenomena during life, but also of those of the structure of the organs after death, with their relations to each other, has greatly advanced our knowledge of pathology, and established numerous and important facts which could never have been obtained by the old school, or Hippocratic mode of investigating disease.

I am satisfied, that no one can read the works of Laennec, Broussais, Andral, Louis, Bouillaud, and a host of other French pathologists; or the works of Abercrombie, Hope, Carswell, Stokes, Gross, and many others among the English and our own countrymen without feeling that his knowledge is increased, and that these works contain numerous facts and observations, unknown to their predecessors, and which could never have been obtained by any observation of the vital phenomena alone, or by any process of *a priori* reasoning, or hypothesis.

But, though it be thus evident that this mode of investigating pathology has vastly increased our knowledge of disease, yet I am far from believing that therapeutics or the treatment of disease has advanced *pari passu* with our pathological knowledge, or that the present mode of treating many diseases derived from observation of symptoms and experience in therapeutical remedies, according to the Hippocratic plan, will ever be benefitted by the subsequent light we may derive from pathological research into the morbid anatomy of the body. What, for example, will it profit us to know the morbid anatomy of an organ, when we know the remedies for the symptoms growing out of this morbid condition, and when we can always recognise the symptoms in other cases, as the same for which we have successfully administered the same remedy? Will any light that may hereafter be thrown upon the morbid anatomy of a child dying of croup ever prevent you from administering an emetic in this disease, which your experience and that of all others, has long ago taught you is a remedy for the symptoms of this disease, which symptoms can always be recognized? Will any development of new pathological conditions of the structure of those dying of intermittent fever ever prevent you from administering quinine as a remedy in this disease? Will any pathological illustrations of the condition of the lungs in pneumonia, or of the pleura in pleurisy, prevent you from bleeding in these diseases? Or will any amount of rigid facts as they are called, emanating from the hospitals of Paris, going to prove that bloodletting is not useful in pleurisy, prevent you from using the remedy; when your own experience and the observation of thousands of practitioners for a long series of years, has fixed it as a remedy which always gives relief? An emetic in croup, quinine in intermittents, and bleeding in pleurisy, are remedies, the value of which, the medical world has tested and proved to be useful. When then we hear it said from Paris there is no *proof* of bleeding being useful in pleurisy, because there is not a regular record of an hundred or more cases, having been treated with and without it, we cannot yield to be cramped to the rule of such rigid nonsense. The experience and observations of thousands prior to the numerical system, has established the efficacy of certain remedies in certain diseases, and no subsequent rules of logic or philosophy can overthrow them. We are far then from believing that the modern mode of cultivating medical science is the *only* true mode. But this is the reasoning of the vitalists, and hence they enquire, wherefore should we be "pottering in the dead-house, and searching in the débis of the body for facts in pathological anatomy, when no knowledge of such facts will enlighten us in our therapeutical indications?" Nay, they contend is it not agreed by the pathological anatomists themselves, that all knowledge both in pathology and therapeutics, is derived only from observation and experience, and that no knowledge of pathology alone teaches us the remedy for the diseased condition? True, this is all so. But here are also the distinctive ideas of the pathological anatomists; they contend that although no knowledge of pathology can instruct us as to the remedy for the diseased condition, yet we can never know all the facts of any pathological state except we examine them by actual observation, both in the living and the dead body, and consequently we can prescribe no remedy with certainty for any disease, without we are acquainted with all the facts in regard to it, which can only be known by a rigid analysis not only of all the symptoms, but of the morbid alterations of structure which produce them; and that in as much as similar symptoms arise from different morbid conditions of the body, it is only by a strict analysis of these, in connection with the condition which produces them, that we can ever arrive at truth. And we may oftentimes be deceived by a mere examination of symptoms alone, when from a knowledge of the different morbid conditions of the organs which may produce them we may approach nearer to the truth. And further, they contend that therapeutics, although not founded upon pathology, can never be advanced with certainty except from a thorough knowledge of pathology. If, for example, we see a disease which from an examination of all the phenomena we recognize as the typhoid disease, and we have learned from observation and experience that a certain remedy is capable of restoring a healthy action to the diseased organs in this disease, then when called to a second case we recognize all the phenomena which characterised the first, we prescribe with confidence and certainty the same remedy which was successful in the first case. But if there is doubt and uncertainty in our diagnosis of the affection, the same doubt and uncertainty must exist with regard to the remedy, and that therefore a correct diagnosis of the true pathological condition is all important to the advancement of practical medicine, for what will it avail us to know that a certain remedy will cure the typhoid disease, when if called to a case of this affection we are ignorant of the phenomena which characterise it; and as already said, how can a knowledge of all the phenomena be obtained except by observation of all the organs of the body and the changes in the vital phenomena?

We are all aware that one of the most difficult parts of our profession is the correct diagnosis of disease, and that we feel relieved and confident of success, or certain of a fatal issue, when we are in possesion of the true pathological condition of our patient. It has been the object of the pathological investigations of the last thirty years to improve our diagnosis of disease, and this, no one will deny, has been accomplished to a very great extent. But, as before observed, our therapeutics have not advanced with our pathological knowledge; when, however, from observation and experience we shall find out the remedy or remedies for a particular diseased condition of the system, the means of diagnosticating this condition being already established by pathological investigations, we are at least in possession of one of the most important elements to the successful advancement of practical medicine. And it must be acknowledged that the slow and doubtful progress of practical medicine for hundreds of years past has been owing, in a very great degree, to the conflicting opinions of medical authors, respecting the true pathology of disease, and to their wild speculations, theories, hypotheses, and systems, the mere creations of ingenious imaginations. At present, however, if in the progress of medical observation a remedy is found to have a controlling influence over a special disease, we are no longer in want of a knowledge of the phenomena by which the same disease can be recognised; and this knowledge is that which has been supplied by modern pathology. But the advancement of therapeutics is even more difficult than that of pathology, and requires a series of observations of the most complex and difficult character, which but few are capable of making, inasmuch as many important considerations not only respecting the true pathology of the disease for which we prescribe, but also the modifying circumstances of age, sex, climate, original consitution, and general condition of the vital organs at the time we prescribe, must all be duly considered and analyzed before we can arrive at truth as to the effect of our remedy. The thousand circumstances which may occur in the ordinary practice of any physician, as the want of time to notice the effects of his remedies, the deceptions practised by the nurse, or the patient himself, the false reports of the effects of the remedy, and the kind interference of some friendly dame with her teas, and drops, and soups which are often administered to gratify the patient, but are never reported except the patient be better, when they generally tell all, together with the intrinsic difficulty of the subject itself, growing out of the extension or diminution of diseased action, independent of any remedy, and the ordinary mode of administering a dozen or more remedies in the course of twenty-four hours, makes it altogether improbable that the general practitioner can ever arrive at anything more, however cautious he may be, than a general conclusion respecting the value of any remedy. And even if he should be successful in a single case of testing the value of a single remedy, he cannot be sure that his observation is correct until he has repeated under different circumstances the same remedy upon different patients, for in the first case the patient may have recovered without the remedy, or even supposing it to be of real value, it may have been by accident administered exactly in the right doses, and at the right time to suit the particular case, and fail in other cases for the want of due discrimination and judgment on the part of the practitioner, as to the quantity, and time of administering it. For it is a fact, that practitioners of medicine may all use the same remedies, and be very unequally successful, not because the remedy used is not equally well adapted to each case, but because it is not properly administered, either as regards time or the amount of the dose adapted to each case. If in a case of simple pleurisy, for example, we direct bleeding to one quart, calomel to 15grs., and a blister 6 inches square, and cure our patient, it does not follow that bleeding to half a pint, calomel to 5grs., and a blister 3 inches square would have had the same effect. So that not only have we to repeat a single remedy in a particular case of disease, but under different circumstances and in different quantities to suit the different cases, before we can be satisfied as to its value. And if this difficulty arises in investigating the value of remedies in the treatment of a disease the pathology of which is known, how much more difficult must it be when we prescribe for a disease the symptoms of which are so complex as to render it doubtful what disease we are prescribing for, or how many organs there are in the system at the time diseased, and to what extent.

The thousand reports of cases, therefore, of which we read every day in the journals, even when given with much apparent exactness respecting all the circumstances, should not inspire us with the belief, that all that is said is true, while we are not justified in rejecting it as false, until we are convinced by subsequent observations and inquiries of our own. Nor should we be too implicitly governed even by the reports of hospital physicians, although in many respects the opportunities they enjoy for correct observations are vastly superior to those of the village or country practitioner. They are liable to err, and the great difficulty of the subject itself is such, that the most extreme caution, untiring labor, and honesty of purpose is required to establish a single fact in therapeutics, or the true value of a single remedy. This difficulty is generally felt and experienced by all enlightened men in the profession, and should warn them to beware how they indiscriminately administer their drugs. The advice of the celebrated Chomel never to do harm with our remedies should ever be present to the mind of the practitioner, and although I believe the cautious and impotent practice of the French schools has failed *directly* to improve the practice of medicine, yet it has given a salutary and extensive check to the indiscriminate system of drugging, which has prevailed to so unlimited and ruinous an extent in our own country.

